# Multiplex Detection of Five Canine Viral Pathogens for Dogs as Laboratory Animals by the Luminex xTAG Assay

**DOI:** 10.3389/fmicb.2018.01783

**Published:** 2018-08-17

**Authors:** Miaoli Wu, Feng Cong, Yujun Zhu, Yuexiao Lian, Meili Chen, Ren Huang, Pengju Guo

**Affiliations:** ^1^Guangdong Provincial Key Laboratory of Laboratory Animals, Guangzhou, China; ^2^Guangdong Laboratory Animals Monitoring Institute, Guangzhou, China

**Keywords:** canine distemper virus, canine parvovirus, canine parainfluenza virus, canine adenovirus, rabies virus, multiplex PCR, xTAG

## Abstract

More and more dogs have been used as a disease model for medical research and drug safety evaluation. Therefore, it is important to make sure that the dogs and their living houses are special pathogen free. In this study, the development and evaluation of a Luminex xTAG assay for simultaneous detection of five canine viruses was carried out, including canine distemper virus, canine parvovirus, canine parainfluenza virus, canine adenovirus, and rabies virus. Assay specificity was accomplished by targeting conserved genomic regions for each virus. Hybridization between multiplexed PCR products and the labeled fluorescence microspheres was detected in a high throughput format using a Luminex fluorescence reader. The Luminex xTAG assay showed high sensitivity with limits of detection for the five viruses was 100 copies/μL. Specificity of the xTAG assay showed no amplification of canine coronavirus, pseudorabies virus and canine influenza virus indicating that the xTAG assay was specific. Seventy-five clinical samples were tested to evaluate the xTAG assay. The results showed 100% coincidence with the conventional PCR method. This is the first report of a specific and sensitive multiplex Luminex xTAG assay for simultaneous detection of five major canine viral pathogens. This assay will be a useful tool for quality control and environmental monitoring for dogs used as laboratory animals, may even be applied in laboratory epidemiological investigations.

## Introduction

Dogs has been extensively used as animal models for medical research and drug evaluation, such as studying human breast cancer carcinogenesis (Abdelmegeed and Mohammed, 2018), Duchenne muscular dystrophy (DMD) (Wells, 2018), management of furcation defect (Afifi et al., 2018), chronic myocardial infarction (MI) (Xiong et al., 2018), comparative pathogenesis study of H3N2 canine influenza virus (CIV) challenged (Luo et al., 2018), long term safety and efficacy of AAV gene therapy (Lee et al., 2018). Therefore, it is critical to make sure that the dogs used as laboratory animals are free of special pathogens. In China, according to the national standard of laboratory dogs, the rabies virus (RV), canine parvovirus (CPV), canine adenovirus (CAV), and canine distemper virus (CDV) are the items that must be tested. All the laboratory dogs and their living houses should be checked up with these viruses regularly for quality monitoring. However, the currently existing detection method recommended by the industry standard is conventional PCR. To complete the whole check routine, four individual PCR should be carried out, which is time-consuming, especially when dealing with a large scale of samples.

A rapid and sensitive multiplex pathogens detection technology is needed to meet the detection requirement of monitoring the animal health status and the sanitary of their living environment. Unlike the existing multiplex PCR methods that only focus on two or three targets and cannot be adapted to high-throughput testing. The luminescent beads are a novel platform for multiplex high-throughput detection and can simultaneously detect up to 100 targets in a single reaction (Dunbar, 2006; Liu et al., 2012). This tool had been adapted for use in genetic analysis, gene expression analysis, SNP analysis, and pathogen identification (Chen et al., 2000; Ocheretina et al., 2013; Zhang et al., 2016).

The Luminex xTAG assay was designed for multiple nucleic acid detection in a single reaction through the hybridization of magnetic microspheres and PCR products (Chen et al., 2015; Reslova et al., 2017). The magnetic microspheres pre-coupled with anti-MagPlex-TAG oligo-nucleotides capture the matching oligo-nucleotide presented on the 5′ end of PCR amplicons. A Luminex fluorescence reader is then used for analytical measurements of bead types. This technology is commercially available for respiratory viral pathogens and gastrointestinal pathogens for human clinical diagnoses (Pabbaraju et al., 2011; Duong et al., 2016), and barely reports on applications in veterinary research.

To fulfill the testing needs of the laboratory dog suppliers for these four viruses and canine parainfluenza virus (CPIV), a multiplex assay for rapid detection of CDV, CPV, RV, CAV, and CPIV in a single tube based on the Luminex xTAG technology was developed and validated.

## Materials and Methods

### Viruses and Vaccines

Canine distemper virus and CPV were obtained from the Institute of Animal Health, Guangdong Academy of Agricultural Sciences (Guangzhou, China). Nucleic acid genomes of canine coronavirus (CCV), pseudorabies virus (PRV), CIV subtype H3N8 and the Nobivac DHPPi vaccine against CDV, CPV, CPIV, and CAV-1 (Intervet, Boxmeer, Netherlands) were preserved by our laboratory. The rabies vaccine and the polyvalent vaccine against CDV, CPV, RV, and CPIV (WuXin Pharmaceutical, Changchun, China) were obtained from the Veterinary Epidemic Prevention Station (Dongguan, China). All viruses, nucleic acids and vaccines were stored at -80°C until use.

Clinical samples were obtained from the local veterinary clinic in Guangzhou, China. DNA and RNA were extracted using a commercial automatic nucleic acid extraction instrument (Tiangen Biotech, Beijing, China) according to the manufacturer’s instructions.

### Multiplex PCR

Viral genomes from the NCBI database were aligned to determine the most conserved genomic regions for each virus. Primers were designed using Primer Premier 5.0 software and inspected with the vector NTI software (**Table 1**). All the forward primers were modified with specific oligo-nucleotide tags that enabled magnetic microsphere conjugation. The 5′ ends of reverse primers were biotinylated to enable detection using streptavidin-R-phycoerythrin (SAPE). All primers and oligo-nucleotides were analyzed with Tag-It Oligo Design Software v.3.00 and purified by high-performance liquid chromatography (Invitrogen, Guangzhou, China).

**Table 1:** Primers used in the multiplex xTAG assay.

Primer	Sequence (5′–3′)	Genome location	Product size (bp)
CDV-F	AACAGATGGGTGAAACAGCA	911~921	97
CDV-R	GCATAACTCCAGAGCAATGGGTAG	984~1007	
CPV-F	CAAGATAAAAGACGTGGTGTAACTC	928~953	123
CPV-R	TTGTGTAGACGCCTCAAAAGAATAA	1026~1050	
CAV-F	TTATGTCTATGGGAACCCTCACTG	2495~2519	135
CAV-R	CGAACACTTCAAACAAAACG	2609~2629	
CPIV-F	TTCTATTCTGCCTACGGATTGTTCT	1215~1234	89
CPIV-R	TCTCTACCCTTTCGATATCAGCTTC	1280~1303	
RV-F	CAGGDCTGGTATCGTTYACTGGGTT	50–75	158
RV-R	GAARTGGRTGAAATAGGARTGAGG	183–207	


The multiplex amplification was performed using a one-step RT-PCR assay kit (QIAGEN, Hilden, Germany) in a total volume of 50 μL as suggested by the manufacturer. The amplification steps were: 50°C for 30 min, 95°C for 15 min and 35 cycles of 94°C for 30 s, 60°C for 30 s, and 72°C for 30 s and a final step of 10 min at 72°C. All samples were tested in triplicate and all assays were run with positive and negative controls.

### Hybridization and Luminex Analysis

MagPlex-TAG microspheres were purchased from Luminex (Austin, TX, United States) and suspended in 1.1× tetramethylammonium chloride as outlined in the manufacturer’s protocol. Nucleic acid hybridization was carried out in a 100 μL volume that included 5 μL of amplified product, 20 μL of the working microsphere mixture that contained 2500 beads of each target-specific microsphere and 75 μL SAPE. Hybridization was carried out at 45°C for 30 min. The products were analyzed using the Luminex 200 reader (Luminex) immediately after hybridization and the results were expressed as mean fluorescence intensity (MFI).

### Specificity and Sensitivity Testing

Pseudorabies virus, CIV, and CCV samples were used as templates in amplification reactions to determine assay specificity. All amplified products were sequenced for verification (Sangon Biotech, Guangzhou, China). Sensitivity testing was carried out using PCR products from CDV, CPV, CAV, RV, and CPIV amplification that were cloned into the pGEM T easy vector (Promega, Madison, WI, United States). CDV, RV, and CPIV were *in vitro* transcribed using T7 RNA polymerase with the RiboMAX Large Scale RNA production kit (Promega) according to the manufacturer’s instructions. RNase-free DNase (Promega) was used to remove plasmid DNA after transcription. TRIzol LS reagent (Invitrogen, Carlsbad, CA, United States) was used for RNA isolation according to the manufacturer’s instructions. Genome copy numbers were calculated based on the nucleic acid concentration and molecular weight. Tenfold serial dilutions of DNA or RNA standards were used as assay templates. All the samples were tested in triplicate.

### Sample Detection

Considering that most of the samples from SPF dogs or their living houses were tested negative, clinical samples from the animal hospital were needed to evaluate the xTAG assay. Clinical samples that included feces, saliva, throat swab, and urine from dogs were tested using conventional PCR (**Table 2**) and the Luminex xTAG assay. Besides, artificial mixed infection samples (by mixing the positive nucleic acid together, or mixing the positive samples together before nucleic acid extraction) were tested for further validation of the xTAG assay. All samples were tested in duplicate. In addition, a comparative test was carried out using the CDV/CPV test card (Quicking Biotech, Shanghai, China) followed by the manufacturer’s instructions.

**Table 2:** Primers used for the conventional PCR method.

Pathogen	Sequence 5′–3′	Amplicon size (bp)
CPV-F	ATCACAGCAAACTCAAGCAGACTT	690
CPV-R	AAATGGTGGTAAGCCCAATGCTC	
CAV-F	TGTGCCCATCGACAAGGAA	433
CAV-R	CTAATAGAAGCGGCCCAACTG	
RV-F	GATCCTGATGAYGTATGTTCCTA	87
RV-R	RGATTCCGTAGCTRGTCCA	
CPIV-F	TACAATCCCACCTACAACA	265
CPIV-R	GAATGATCCCTCCTCAAAGC	
CDV-F	GCTTACTTCAGACTCGGGCAAGAAATGGTTA	154
CDV-R	CAGTAGCTCGAATTGTCCGGTCCTCTGTTGT	


### Data Analysis and Cutoff Definition

Mean fluorescence intensity values were calculated using software supplied with the Luminex reader after counting at least 100 beads for each bead set in a sample (Ponzoni et al., 2013). Background calculations used blank controls containing all the hybridization components except target DNA or RNA. The cutoff value of each bead set was defined as the net MFI value of negative controls +3 SD as previously defined (Dangoudoubiyam et al., 2011).

## Results

**FIGURE 1:**
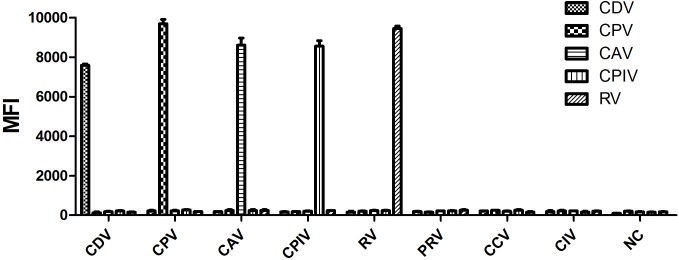
Specificity analysis of the 5-plex Luminex xTAG assay. Each bar represents the average MFI of duplicate samples. CDV, canine distemper virus; CPV, canine parvovirus; CAV, canine adenovirus; CPIV, canine parainfluenza virus; RV, rabies virus; PRV, pseudorabies virus; CCV, canine coronavirus; CIV, canine influenza virus; NC, negative control (water). The result of the 5-plex Luminex xTAG assay showed high specificity.

Nucleic acid of PRV, CIV, and CCV were used as templates in this study to evaluate the specificity of the multiplex-PCR primers. The results showed that, all primers pairs were found to detect their specific viral target with no amplification of non-target samples. The fluorescence signals were observed only in the positive controls (**Figure 1**), revealing a good specificity of the assay.

**FIGURE 2:**
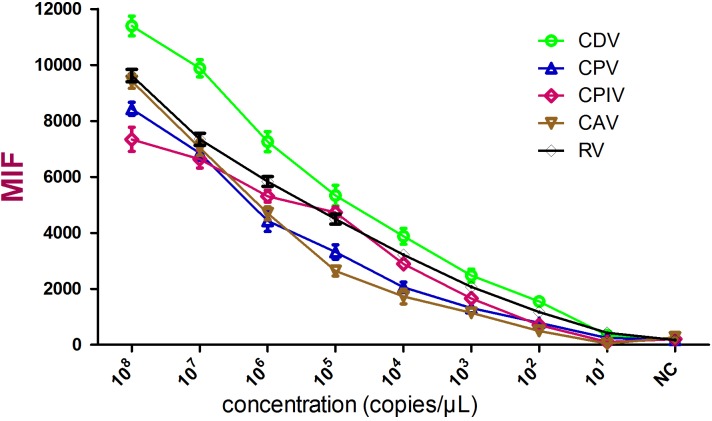
Sensitivity analysis of the xTAG assay. Cloned gene copies from the indicated viruses were transcribed *in vitro* and used in triplicate. The detection limit was 100 copies/μL for these five viruses.

Sensitivity was tested using 10-fold serial dilutions of standards and the limit of detection was defined as the highest dilution that resulted in an MFI value above the cutoff value. In this study, these five viruses shared different cutoff values, which were based on the MFI values of their own negative controls. Data analysis showed that the limit of detection for each of the five viruses was about 100 copies/μL (**Figure 2** and **Table 3**).

**Table 3:** MFI values of different recombinant plasmid concentrations.

Sample (copies/μL)	CDV	CPV	CPIV	CAV	RV
10^8^	11394.5	8122.5	7085.5	9012.5	9619.6
10^7^	9883	6535	6634.5	6944.5	7342
10^6^	7262	4432	5311	4699	5849
10^5^	5335	3319	4733	2638.5	4440.5
10^4^	3879.5	2056	2891.5	1730.5	3214
10^3^	2475	1312	1661	1144	2075
10^2^	1552.5	791	707	497.5	1174
10^1^	348	247	106	47.7	431


The reproducibility and stability of the assay was determined using parallel reactions at concentrations of 10^8^, 10^5^, and 10^3^ copies/μL of standard DNA/RNA. The inter-assay variance was less than 3%, while the intra-assay variance was less than 4%. The low inter-assay and intra-assay coefficient of variations demonstrated a high repeatability of the xTAG assay (**Table 4**).

**Table 4:** Reproducibility and stability analysis of the Luminex xTAG assay.

Virus	Copies/μL	Intra-assay MFI			CV%	Inter-assay MFI			CV%
		1	2	3		1	2	3	
CDV	10^8^	10286	10723	10488	2.08	11501	10864	10711	3.80
	10^5^	5429	5623	5466	1.87	5418	5589	5703	2.57
	10^3^	2485	2395	2501	2.32	2386	2475	2529	2.93
CPV	10^8^	8468	8153	8361	1.92	8218	8575	8165	2.68
	10^5^	3198	3396	3256	2.89	3295	3188	3426	3.61
	10^3^	1353	1298	1295	2.48	1346	1286	1285	2.67
CAV	10^8^	7399	7291	7101	2.07	7047	7245	7435	2.68
	10^5^	2719	2693	2596	2.42	2548	2683	2714	3.33
	10^3^	1129	1167	1189	2.61	1135	1205	1145	3.26
CPIV	10^8^	9225	9464	9048	2.25	9370	9167	9186	1.21
	10^5^	4849	4704	4627	2.38	4688	4754	4801	1.20
	10^3^	1663	1692	1630	1.87	1621	1684	1673	2.03
RV	10^8^	9634	9714	9511	1.06	9743	9812	9569	1.28
	10^5^	4483	4513	4326	2.26	4512	4643	4486	1.85
	10^3^	2008	2093	1984	2.82	2043	1994	2063	3.94


A total number of 75 clinical samples were evaluated by both the Luminex xTAG assay and conventional PCR. The positive rates for CDV, CPV, CAV, and CPIV were 8% (6/75), 50.7% (38/75), 2.7% (2/75), and 4% (3/75), respectively. No sample tested positive for RV and no mixed infections were identified. The detection results of these two methods were in 100% agreement. The positive detection rate of CDV test card was 8% (6/75), which was fully consistent with the xTAG assay. However, only 35 samples were tested positive with the CPV test card, three positive samples (confirmed by sequencing) were falsely detected as negative. To a certain extent, the xTAG assay was more sensitive than the colloidal gold test strip, as revealed by the comparison results (Supplementary Table S1).

**FIGURE 3:**
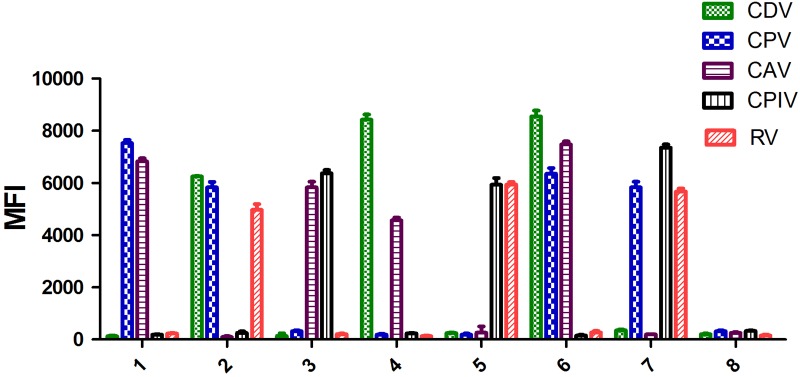
Validation of the artificial mixed infection samples. 1, CPV and CAV positive sample; 2, CDV, CPV, and RV positive sample; 3, CAV and CPIV positive samples; 4, CDV and CAV positive sample; 5, CPIV and RV positive sample; 6, CDV, CPV, and CAV positive sample; 7, CPV, CPIV, and RV positive sample; 8, negative control.

To determine the utility of the multiplex xTAG assay in multiplex infection, artificial mixed infection samples were tested. Three samples were found triple infection and four samples were found double infection (**Figure 3**). Indicating that, the new developed multiplex xTAG assay had the ability to identify different targets in co-infection samples.

## Discussion

The multiplex PCR format allows for the simultaneous detection of different nucleic acid sequences in a single reaction. This is time- and labor-saving, but the interpretation of conventional multiplex PCR relies on amplicon size, restricting its use in a high-throughput format (Piewbang et al., 2017). The Luminex xTAG platform was used in this study to develop specific and sensitive high-throughput assays that can be expanded to include more pathogens as needed (Jiang et al., 2017). This format has been widely used in human disease diagnosis but not available in dogs (Wessels et al., 2014; Dunbar et al., 2015; Laamiri et al., 2016).

Dogs are important experimental animals with strict quality control. CDV, CPV, CAV, and RV are the pathogens that must be excluded according to the national standard of laboratory animal in China. The xTAG technology was used to develop high throughput assays for the rapid and sensitive simultaneous detection of these viruses to help with laboratory dog’s daily monitoring.

In this study, a 5-plex Luminex xTAG assay for the simultaneous detection of CDV, CPV, CPIV, CAV, and RV in a single reaction was developed and validated. This is the first report of a Luminex xTAG assay for use in canine pathogen identification, and it’s expected to replace the currently existing single PCR method for canine daily monitoring. However, this panel may not be a good tool for clinical diagnosis, compared with the fast and easy point-of-care testing (POCT).

Primer design is the key for the establishment of Luminex xTAG assay. Unwanted hairpin structures would increase the MFI background. The bio-informatics analysis was applied to evaluate specificity and avoid primer dimers. Specific primers targeted conserved genomic regions for each virus were selected. This approach yielded an assay without cross-reactions between target and non-target viruses.

The limit of detection for the multiplex Luminex xTAG assay was estimated using viral DNA/RNA generated from cloned viral fragments. The assay sensitivity was 100 copies/μL, which might be slightly lower than real-time PCR (Schlottau et al., 2017). Validation of 75 clinical samples revealed a 100% coincidence rate with conventional PCR detection, indicating that the assay was sufficient for the detection of these five pathogens in clinical samples. Artificial mixed infection samples were verified, revealing that the assay was capable of identifying specific targets in co-infection samples.

In this study, a novel multiplex platform for the simultaneous detection of five important canine viral pathogens was established. This assay will be useful for animal health status and their living house monitoring. Additional targets can be integrated into the existing multiplex platform as needed and allows increased flexibility in disease investigation and environmental monitoring.

## Author Contributions

MW carried out data analysis and manuscript writing. FC, YZ, and YL participated in manuscript preparation. MC, RH, and PG designed the experiments. All authors read and approved the final manuscript.

## Conflict of Interest Statement

The authors declare that the research was conducted in the absence of any commercial or financial relationships that could be construed as a potential conflict of interest.
